# Diphtheria outbreak in Somalia: a weekly sitrep on the recent health crisis-2025

**DOI:** 10.1186/s41182-025-00843-0

**Published:** 2025-11-19

**Authors:** Saadaq Adan Hussein, Marian Muse Osman, Mohamed Mohamoud Hassan, Yahye Sheikh Abdulle Hassan, Abdirahman Aden Hussein, Rage Adem, Mohamed M. Ali Fuje, Ayan Nur Ali, Abdinur Hussein Mohamed, Khadar Hussein Mohamud, Abdirahman Moallim Ibrahim, Mohamed Farah Yusuf, Abdinur Adan Hussein, Abdullahi Mohamed Mohamud, AbdulJalil Abdullahi Ali

**Affiliations:** 1 Department of School of Postgraduate Studies, Benadir University, Hodan Benadir, Mogadishu, Somalia; 2 Department of Social and Human Capital Development Pillar, Office of the Prime Minister, Federal Republic, Mogadishu, Somalia; 3 Department of Research and Policy Development, SOR Institute: Somalia Social Research, Mogadishu, Somalia; 4 Department Benadir Institute for Research and Development, Benadir University, Mogadishu, Somalia; 5 Department Research, Somali National Institute of Health, Mogadishu, Somalia; 6 Department Office Rector at, Benadir University, Mogadishu, Somalia; 7 Department of Medicine and Surgery, Jamhuriya University of Science and Technology, Mogadishu, Somalia; 8 Department Innovation Hub, Benadir University, Mogadishu, Somalia; 9 Department of Emergency, Mogadishu Somali Türkiye Training and Research Hospital, Mogadishu, Somalia; 10 Department Somali Development Research Institute (SODRI), Mogadishu, Somalia; 11 Department Faculty of Medicine and Surgery, Jazeera University, Mogadishu, Somalia; 12 Department of CEO, Tayo Institute for Research and Development, Mogadishu, Somalia; 13 Department of Civil Engineering at Amoud University, Borama, Somalia

**Keywords:** Diphtheria, Corynebacterium, Somalia, Outbreak, Vaccination, Public, Health, Crisis

## Abstract

The global diphtheria incidence has fallen following widespread use of the diphtheria–tetanus–pertussis (DTP) vaccine; pockets of low coverage and disrupted health services continue to fuel outbreaks. Somalia, already challenged by conflict-related displacement and fragile health infrastructure, declared a national diphtheria outbreak on 19 August 2025. We analysed weekly case-based surveillance data reported through Somalia’s Integrated Disease Surveillance and Response (IDSR) system and the DHIS2 electronic platform (Epi-weeks 1–33, 2025) on the platform. Vaccination-coverage trends were extracted from WHO/UNICEF Estimates of National Immunization Coverage (WUENIC, 2000–2024). Supplementary information was obtained from the Ministry of Health situation reports and partner briefs. By Epi-week 33 (ending 17 August 2025), 1811 suspected diphtheria cases (17 laboratory-confirmed) and 89 deaths were recorded (case-fatality rate 5%). Children < 5 years accounted for 56% of cases; 87% of patients had no documented diphtheria immunization. Weekly incidence accelerated sharply after Epi-week 20, with the largest surges in Puntland, South-West State and the Benadir Regional Administration. DTP-1 coverage increased from 40 to 60% (2000–2018) to 79% in 2022 but plateaued at 70% in 2024; DTP-3 coverage reached 71% in 2022 yet remains insufficient for herd protection. Despite targeted ring vaccination and distribution of diphtheria antitoxin (DAT), constrained vaccine and DAT supplies, insecurity, and access barriers hamper outbreak control. Somalia’s diphtheria resurgence underscores how conflict, displacement, and uneven immunization can reverse hard-won gains against vaccine-preventable diseases. Closing routine-coverage gaps, guaranteeing timely DAT and antibiotic access, expanding real-time surveillance, and intensifying community engagement are urgent priorities to halt transmission and avert additional deaths. Prompt mobilization of national leadership, donors, and technical partners is essential to contain the outbreak and restore progress toward diphtheria elimination.

**To the editor,**

Diphtheria was once a leading cause of childhood mortality until vaccination became widespread. Following the roll-out of the diphtheria tetanus pertussis (DTP) vaccine initially in high-income countries after World War II and, from 1974, through the World Health Organization’s Expanded Programme on Immunization in lower-income nations global case counts plummeted [1]. Although universal immunization brought the disease under control in the former Soviet Union, large epidemics resurged in the 1990s across the Newly Independent States [2]. Today, diphtheria remains a public-health threat where routine coverage is suboptimal, with outbreaks reported in sub-Saharan Africa and Asia and, sporadically, even in well-vaccinated regions such as Europe and the Middle East [3]. In the WHO African Region, active outbreaks are documented in Guinea, Mauritania, Niger, Nigeria and South Africa, while vulnerable countries—including Somalia—remain at high risk of further transmission [4].

Despite the steep global decline in diphtheria since the advent of routine immunization, the disease has not been eliminated. In 2021, 8639 cases were reported worldwide, more than half (51.6%) of them in Ethiopia. Resurgences persist where vaccination coverage is patchy, and health systems are disrupted; recent outbreaks in Pakistan, Yemen and India illustrate how population movements and fragile services can reignite transmission [5, 6]. In Somalia, the threat is amplified for internally displaced communities, who often live far from clinics reachable only via unsafe roads; insecurity and seasonal floods further restrict health-worker access. These gaps place already vulnerable groups at heightened risk and underscore the need to strengthen routine immunization, surveillance and delivery systems alongside humanitarian support [7].

Diphtheria’s lethality is driven by systemic absorption of its exotoxin. Myocarditis, malignant arrhythmias, acute renal failure and peripheral neuropathies are the most feared complications; without prompt antitoxin and antibiotic therapy, toxin-mediated damage can progress rapidly, and myocarditis or polyneuropathy often proves fatal. Cutaneous infection, typically a chronic ulcer coated with a grey pseudomembrane, constitutes an under-recognized reservoir of transmission in crowded or displaced settings where vaccination gaps already fuel respiratory outbreaks [6, 8]. Overall, the case-fatality rate ranges from 5 to 17%, rising steeply among children under five and adults over 40, a sobering statistic that underscores the urgency of closing immunization and treatment gaps highlighted above [9].

Somalia officially declared a diphtheria outbreak on 19 August 2025, issuing an epidemiological alert after a steep, multi-regional surge in cases and deaths in a country already marked by mass displacement and chronically low vaccination coverage. According to Hussein Abdukar Muhidin, Director-General of the National Institute of Health, transmission has expanded to “an unprecedented level” that warrants immediate action [10–12]. Case definition in this outbreak was based on WHO-approved [13] case definitions for outbreaks: suspect (clinical criteria only), probable (clinical and with epidemiological link) and confirmed (laboratory proved) cases. Seventeen laboratory-confirmed cases were diagnosed through RT‑PCR testing of pharyngeal swab specimens at Somalia’s National Public Health Reference Laboratory. The remaining probable and suspect cases were clinically categorized according to pseudomembrane formation and epidemiological linkage during outbreak clusters. These definitions facilitate comparison with WHO and CDC surveillance requirements, and enable uniform international reporting of diphtheria cases.

National Surveillance data through the national IDSR system (Epi-Week 33, ending 17 August 2025) show a total of 1,811 suspected diphtheria cases and 89 deaths Among these, 17 (35.4%) cases were microbiologically confirmed by reverse transcription-polymerase chain reaction (RT-PCR) for Corynebacterium diphtheriae at the National Public Health Reference Laboratory (NPHRL), Mogadishu, a fourfold increase over the same period in 2024 with an overall case-fatality rate of 5%. Children under five account for 56% of cases, and 87% of all patients report no prior diphtheria immunization, underscoring the impact of fragile routine services in heavily displaced areas such as Banadir, Puntland and Galmudug. Through the only national capacity to have Somalia as National Public Health Reference Laboratory NPHRL Somalia, confirmation by RT-PCR has identified 17 Corynebacterium diphtheria-positive samples, and 22 community deaths have been recorded outside health-facility settings (Fig. 1) [14, 15]. The low number of confirmed cases is, to some extent, a reflection of the national capacity to diagnose which is itself limited and further hindered by difficulties in sample transportation from both remote and insecure areas. The establishment of regional microbiology hubs in order to provide input into outbreak response and toxigenicity confirmation will be critical in the future, as will the introduction of validated rapid molecular assays.

**Fig. 1:**
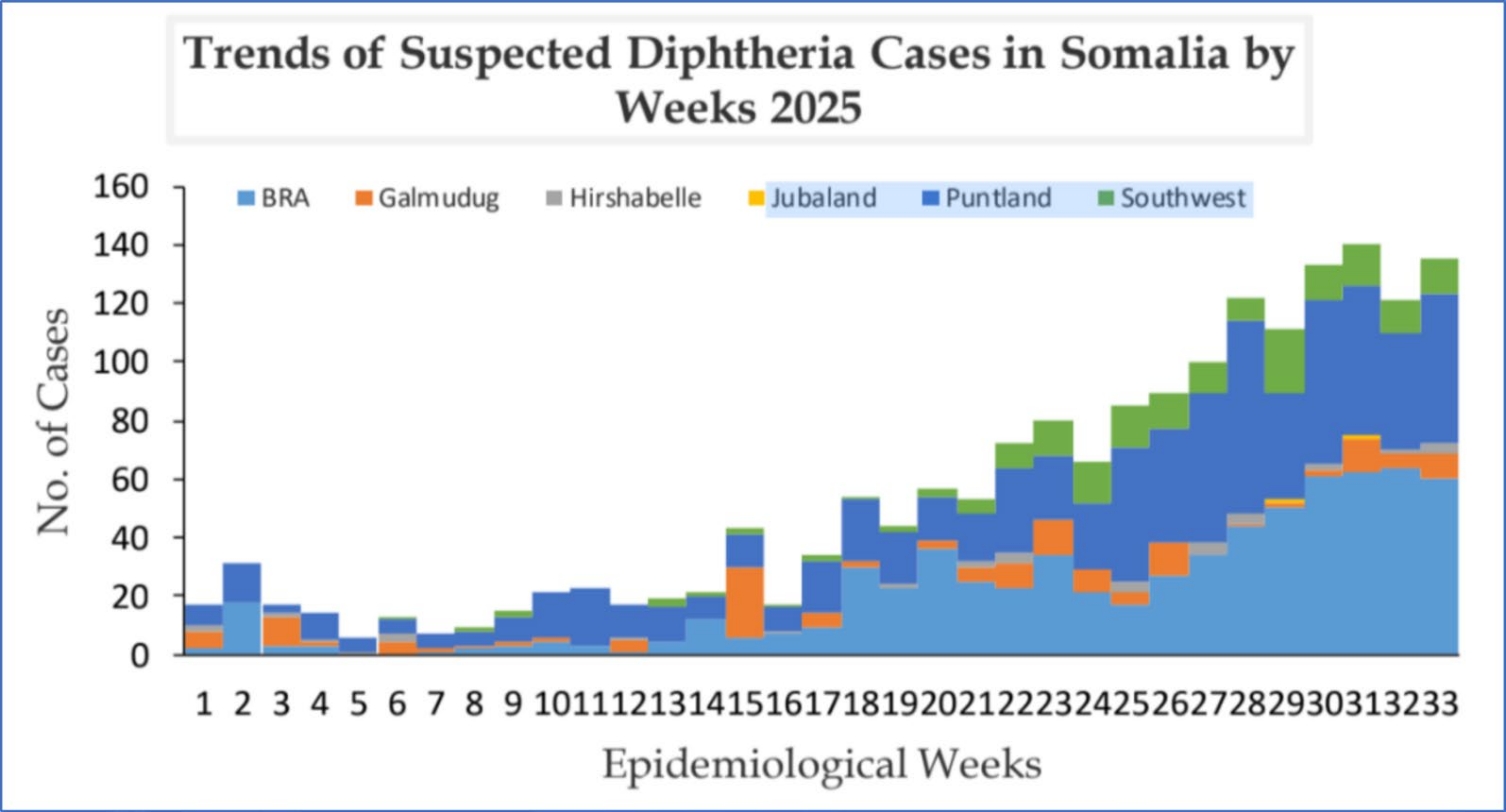
Weekly distribution of suspected diphtheria cases in Somalia by region, epidemiological weeks 1–33, 2025

Epidemiological curves reveal a sharp acceleration after Epi-Week 20, driven largely by spikes in Puntland, the South-West State and the Benadir Regional Administration. Despite persistent humanitarian constraints, the Integrated Disease Surveillance and Response (IDSR) system strengthened through WHO collaboration and deployment of the DHIS2 real-time reporting online platform has enabled early case detection and situational awareness [16]. These data highlight an urgent need to close vaccination gaps, expand antitoxin access and bolster frontline surveillance to curb Somalia’s rapidly evolving outbreak. Routine immunization has improved in Somalia but remains insufficient to curb the current outbreak. DTP-1 coverage rose from 40 to 60% during 2000–2018 to 65% in 2019, peaking at 79% in 2022; DTP-3 climbed from 26 to 33% in the early 2000s to 71% in 2022 and has plateaued near 70% through 2024 (Fig. 2). Yet immunity gaps persist particularly among displaced communities allowing an outbreak first detected in Mahaday district, Hir-Shabelle, to spread across Banadir, Puntland and Galmudug [17].

**Fig. 2:**
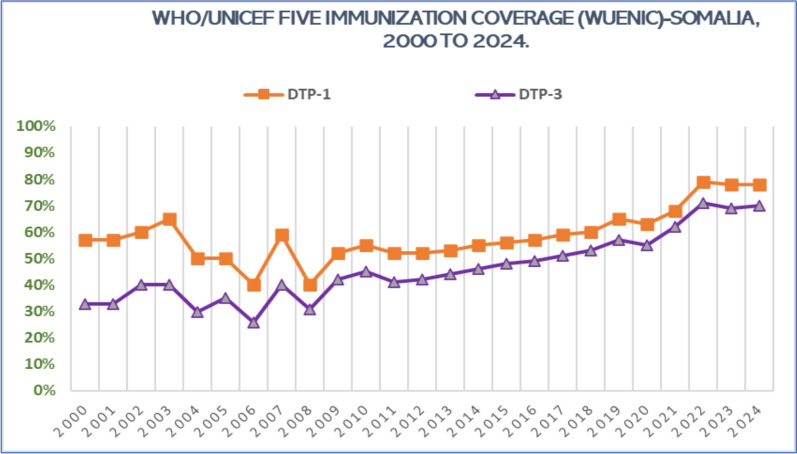
Trends in WHO/UNICEF estimated immunization coverage (WUENIC) for DTP-1 and DTP-3—2000 to 2024

The Ministry of Health, supported by the World Health Organization (WHO) and UNICEF, is running supplemental campaigns: pentavalent vaccine for children under five and tetanus–diphtheria (Td) for those aged five and above. Parallel case-management efforts provide diphtheria antitoxin (DAT) and supportive care, but stock shortages limit coverage. Intensified DHIS2-based surveillance recorded an 11.5% increase in cases between epidemiological weeks 32 and 33 of 2025, underlining continued transmission [14, 15]. Sustained control will require additional vaccines, DAT, medical supplies and trained personnel to close coverage gaps and stem further spread [18].Timely administration of diphtheria antitoxin (DAT) and antibiotics is critical for severe-case management, yet the outbreak’s continued expansion shows that treatment alone cannot halt transmission. WHO and the Ministry of Health have trained more than 150 clinicians in DAT use and are running ring-vaccination drives in high-risk districts, but constrained supplies and fragile logistics still slow the delivery of antitoxin, medicines and vaccines to remote areas [19, 20].

Somalia’s Field Epidemiology Training Programme (FETP) is bolstering the response by equipping local professionals with skills in surveillance, case investigation and outbreak control; its graduates now serve on rapid-response teams, accelerating detection and containment of new clusters [21]. Recent vaccine donations channelled to displacement camps and other vulnerable settings are widening coverage of the ongoing pentavalent and tetanus–diphtheria campaigns. Sustained support from WHO, UNICEF and other partners, alongside additional resources for vaccines, DAT and trained personnel, is essential to scale up immunization and bring the outbreak under lasting control [18, 22].Somalia’s escalating diphtheria outbreak constitutes a public-health emergency supercharged by conflict, mass displacement and chronically fragile services. Curbing further morbidity and mortality demands an urgent, coordinated response: national authorities, donors and partners must close routine-immunization gaps, secure a reliable supply of diphtheria antitoxin and other essential medicines, expand safe access to care in hard-to-reach areas and mobilize communities to build trust in vaccination and early treatment. Without swift, well-resourced action, the disease will continue to spread among the country’s most vulnerable populations, claiming avoidable lives [23].

Outbreak containment in Somalia was based on the triad approach: early case detection, active contact tracing and post-exposure prophylaxis for confirmed contacts. Contact tracing within 24 h was effective at preventing community spread, and antibiotic prophylaxis with erythromycin or benzathine penicillin G lowered the risk of carriage. The use of booster vaccination by ring vaccination in the households and schools within a 1 km diameter around cases provided an opportunity for complete reinforcement of immunity. Yet, there are gaps in terms of turnaround time for lab confirmation and confirmation through follow-up culture, which highlights the necessity to decentralize the diagnostic capability.

## Discussion

Despite confirmatory testing of suspected cases being performed effectively with RT-PCR at the National Public Health Reference Laboratory, Somalia currently has no in-country toxigenicity assays, e.g. Elek test. Additionally, the lack of regional molecular testing hubs further prolongs the time taken to receive diagnoses with samples being collected from remote communities. Decentralization of RT-PCR capacity and facilitation of toxigenicity confirmation by regional or international reference laboratories are needed to improve diphtheria surveillance and response in Somalia.

The current outbreak is part of a general trend of re-emergence that has been recently described for conflict-affected and low-immunization countries. Close to 900 cases were recorded in previous outbreaks (2023–2024) in Somalia, but the current upsurge in 2025 has confirmed and probable cases exceeding 1600 with 87 fatalities [24]. Relatively explosive outbreaks have also unfolded in Yemen (1671 suspected cases as of 2023) [25], Nigeria (10,609 suspected between 2023 and 2024) [26], illustrating the broader malaise that exists with regard to vaccine access, availability of antitoxins and capacity for diagnosis within fragile health systems. These comparisons emphasize the urgency of consistent immunization coverage, timely case confirmation and regional antitoxin stockpiling to reduce mortality and avoid repeated epidemics.

## Recommendation

### Strengthen vaccination campaigns

The Ministry of Health (MOH), supported by WHO and UNICEF, should urgently scale up immunization efforts, especially in high-risk areas and displaced communities. This includes targeting children under five for pentavalent vaccinations and providing catch-up vaccinations for children aged 5–15. Community outreach programmes should be implemented to raise awareness about timely immunization, with clear coverage targets set for affected regions.

### Enhance surveillance and reporting systems

The Ministry of Health (MOH) should expand the implementation of the Integrated Disease Surveillance and Response (IDSR) system across all districts, rapid diagnostic tests to utilize the Field Epidemiology Training Program (FETP). This approach will facilitate early detection, timely and a swift response to emerging cases, particularly in remote or conflict-affected regions. Surveillance teams, working in coordination with local health workers, should prioritize timely reporting and real-time data analysis to ensure an effective and responsive outbreak control strategy.

### Improve case management and medical supplies

The MOH, in collaboration with international health organizations, must ensure an uninterrupted supply of Diphtheria Antitoxin (DAT) and antibiotics. In parallel, healthcare workers should receive ongoing training in case management, including the use of DAT, with regular workshops to maintain high standards of care across affected districts.

### Strengthening community engagement and risk communication

The MOH, working with local leaders and media outlets, should launch comprehensive risk communication campaigns. These campaigns must include radio broadcasts, mobile messaging, and community meetings to educate the public on diphtheria prevention and early treatment. The goal is to increase public awareness and ensure behaviour changes in the next 3 months.

### Increase international support

The MOH must continue to engage with global health organizations and donors to secure financial and logistical support for the outbreak response. This includes the rapid deployment of medical supplies, vaccines, and trained personnel to the affected regions, as well as strengthening local healthcare infrastructure to ensure sustained response efforts.

## Conclusion

Somalia’s diphtheria resurgence epitomizes how conflict, displacement and fragile health systems can unravel decades-long gains in vaccine-preventable disease control. Rapidly rising case counts, high fatality among undervaccinated children and persistent supply-chain gaps demonstrate that neither treatment nor reactive campaigns alone will suffice. Only a fully resourced, multisectoral effort—one that closes routine-immunization gaps, guarantees timely access to antitoxin and antibiotics, strengthens real-time surveillance, and earns public trust through targeted risk communication—can halt transmission and prevent further loss of life. Prompt mobilization of national leadership, donor funding and technical partners is therefore imperative to protect Somalia’s most vulnerable populations and restore momentum toward diphtheria elimination.

## Data Availability

No datasets were generated or analysed during the current study.
